# Fe65-PTB2 Dimerization Mimics Fe65-APP Interaction

**DOI:** 10.3389/fnmol.2017.00140

**Published:** 2017-05-11

**Authors:** Lukas P. Feilen, Kevin Haubrich, Paul Strecker, Sabine Probst, Simone Eggert, Gunter Stier, Irmgard Sinning, Uwe Konietzko, Stefan Kins, Bernd Simon, Klemens Wild

**Affiliations:** ^1^Heidelberg University Biochemistry Center (BZH), University of HeidelbergHeidelberg, Germany; ^2^European Molecular Biology Laboratory (EMBL), Structural and Computational BiologyHeidelberg, Germany; ^3^Division of Human Biology and Human Genetics, University of KaiserslauternKaiserslautern, Germany; ^4^Institute for Regenerative Medicine (IREM), University of ZurichZurich, Switzerland

**Keywords:** Fe65, phosphotyrosine-binding domain (PTB), homodimerization, amyloid precursor protein (APP), AICD, Alzheimer’s disease

## Abstract

Physiological function and pathology of the Alzheimer’s disease causing amyloid precursor protein (APP) are correlated with its cytosolic adaptor Fe65 encompassing a WW and two phosphotyrosine-binding domains (PTBs). The C-terminal Fe65-PTB2 binds a large portion of the APP intracellular domain (AICD) including the GYENPTY internalization sequence fingerprint. AICD binding to Fe65-PTB2 opens an intra-molecular interaction causing a structural change and altering Fe65 activity. Here we show that in the absence of the AICD, Fe65-PTB2 forms a homodimer in solution and determine its crystal structure at 2.6 Å resolution. Dimerization involves the unwinding of a C-terminal α-helix that mimics binding of the AICD internalization sequence, thus shielding the hydrophobic binding pocket. Specific dimer formation is validated by nuclear magnetic resonance (NMR) techniques and cell-based analyses reveal that Fe65-PTB2 together with the WW domain are necessary and sufficient for dimerization. Together, our data demonstrate that Fe65 dimerizes via its APP interaction site, suggesting that besides intra- also intermolecular interactions between Fe65 molecules contribute to homeostatic regulation of APP mediated signaling.

## Introduction

The Fe65s (Fe65, Fe65L1 and Fe65L2) are a family of conserved eukaryotic adaptor proteins involved in a variety of biological processes (Russo et al., 1998; McLoughlin and Miller, 2008; Minopoli et al., 2012). Special attention has been given to the brain-enriched Fe65 as its expression pattern parallels the amyloid precursor protein (APP; Guenette et al., 2006). Accordingly, the physiological functions of the two proteins are interdependent and knockout studies resulted in markedly similar phenotypes (Zambrano et al., 2002; Guenette et al., 2006; Strecker et al., 2016). APP is a single-spanning type-1 transmembrane protein (Coburger et al., 2014) with numerous neuronal functions especially in the developing brain (Müller and Zheng, 2012). Sequential regulated proteolysis of APP by different secretases (Lichtenthaler et al., 2011; Haass et al., 2012) results in multiple break-down products including soluble ectodomains, the Aβ-peptides forming the amyloids in Alzheimer’s disease, and the APP intracellular domain (AICD) that is released into the cytosol (Selkoe and Hardy, 2016). The AICD is an intrinsically disordered peptide of 47 residues (Ramelot et al., 2000) and includes the GYENPTY internalization sequence that besides Fe65 binds also to many other adaptor proteins (Russo et al., 1998) with a variety of physiological functions and pathological implications (Müller et al., 2008; Pardossi-Piquard and Checler, 2012).

**Figure 1:**
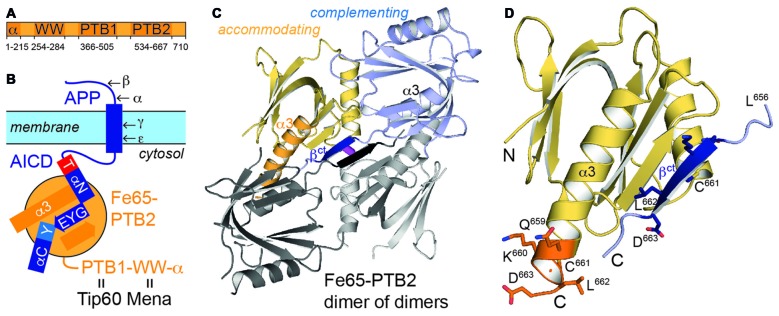
**Fe65 and amyloid precursor protein (APP). (A)** Domain architecture of human Fe65 with numbering of domain boundaries. **(B)** Schematic for Fe65-mediated APP-signaling by the APP intracellular domain (AICD)/Fe65-phosphotyrosine-binding domains 2 (PTB2) complex at the cell membrane. Structural details for the interaction are depicted as follows: αN and αC: AICD helices; T and Y: AICD sequence fingerprints (T: T^668^PEE, Y: N^684^PTY) as part of AICD helices, GYE: AICD region involved in β-augmentation with Fe65-PTB2. APP-cleavage sites by secretases are indicated by Greek symbols. **(C)** X-ray structure of the Fe65-PTB2 dimer of dimers. The dimer is constituted by a “complementing” subunit (blue) with a transition of the C-terminus to strand β^ct^ (dark blue), while the “accommodating” subunit (yellow) contains the entire helix α3 (orange). The second dimer symmetrically attached by β-augmentation is shown with gray subunits. The central disulfide bond connecting the dimer of dimers is shown in magenta. **(D)** Close-up on the C-terminal Fe65-transition. According regions (L^656^-D^663^) of the complementing (dark blue, β^ct^) and accommodating (orange, α3) subunits are given with side chains and numbering.

Fe65 determines localization and nuclear signaling of APP and modulates APP processing and Aβ-peptide generation (McLoughlin and Miller, 2008). Fe65 is a multidomain protein including an N-terminal α-helical domain and three protein-protein interaction modules: a WW domain and two consecutive C-terminal phosphotyrosine-binding (PTB) domains (Figure 1A). The WW domain binds to the Mena protein (Ermekova et al., 1997) involved in actin dynamics and cell motility thus regulating neuronal positioning in the developing brain. Fe65-PTB1 has been mainly implicated as central module of a ternary AICD/Fe65/Tip60 complex responsible for transcriptional activity of APP (Cao and Südhof, 2001), with the histone acetyltransferase Tip60 being a key regulator of genome expression and stability. Further data suggested Fe65 to provide a dominant role for nuclear signaling (Yang et al., 2006). The analysis of the AICD/Fe65/Tip60 interaction revealed that only membrane-bound AICD in context of APP and not on its own is a potent transactivator of transcription (Cao and Südhof, 2004). The distinction had been interpreted by a membrane association dependent transition of Fe65 from a closed to an open and active conformation, involving its WW and PTB2 domain.

Most attention has been given to Fe65-PTB2 as it directly interacts with the AICD and thus functionally joins the two proteins (Borg et al., 1996). The interaction is phosphotyrosine-independent and untypically for PTB-interactions (Uhlik et al., 2005) includes an extended interface of 28 AICD residues including two α-helices (αN and αC; Figure 1B; Radzimanowski et al., 2008c). The GYENPTY internalization sequence is recognized in a rather hydrophobic crevice with GYE involved in a PTB-typical β-augmentation manner and NPTY starting helix αC and placing the canonical PTB-relevant tyrosine in its binding pocket. Unique for the AICD/Fe65-PTB2 complex is the N-terminal binding helix αN within AICD that is capped by the T^668^PEE-motif. Phosphorylation of threonine T^668^ regulates the interaction and has been identified as sensitive checkpoint switching between physiological and pathological APP related pathways (Ando et al., 2001).

Here we present structural and functional data on Fe65-PTB2 revealing the domain as flexible module forming a homodimer *in vitro* and *ex vivo* in the absence of APP. Dimerization mimics the AICD-interaction and at the same time shields the hydrophobic crevice. The interaction competes with AICD binding and therefore with APP signaling depending on its cellular context.

## Materials and Methods

### Protein Production and Characterization for X-ray Structure Analysis

Human Fe65-PTB2 (residues 534-667; UniPROTKB: APBB1_HUMAN, O00213) was expressed and purified for crystallization as described previously (Radzimanowski et al., 2008a). To avoid precipitation of concentrated and pure Fe65-PTB2, 5% (v/v) glycerol was added in the final size exclusion chromatography (SEC) buffer. Multi angle light scattering (MALS) was performed in line with SEC and monitored by refractive index measurements (Wyatt technology). The protein (5–20 mg/mL) was crystallized within 3 days in an automated platform at 18°C by mixing equal amounts (200 nL) of protein solution and a reservoir containing 1.6 M ammonium sulfate, 0.08 M sodium acetate pH 4.6 and 20% (v/v) glycerol in a sitting drop setup. The high glycerol concentration allowed direct flash-cooling in liquid nitrogen for X-ray structure analysis. X-ray data collection was done at beamline ID29 of the European Synchrotron Radiation Facility (ESRF). Data was integrated with program XDS (Kabsch, 2010) and scaled and merged with program AIMLESS (Evans and Murshudov, 2013) from the CCP4-package (Winn et al., 2011). The structure was solved by the Molecular Replacement method (PHENIX package; Adams et al., 2010) using a monomeric Fe65-PTB2 molecule taken out of the Fe65-PTB2/AICD complex (PDB entry: 3dxc). Iterative model building, refinement and validation were performed with programs COOT (Emsley et al., 2010) and PHENIX. All structural figures were prepared using PyMOL (Molecular Graphics System, Version 1.5.0.4 Schrödinger, LLC)^1^.

### NMR Measurements

Sequences for wildtype (wt) Fe65-PTB2 and the C633E mutant were cloned into a pETHis vector using NcoI/BamHI restriction enzymes. The proteins were expressed in *E. coli* BL21(DE3) Rosetta pLysS grown in LB media or for ^15^N- or ^13^C/^15^N-labeling in M9 media by induction with 0.5 mM IPTG overnight at 22°C. Pellets were lysed by sonication in 20 mM Tris pH 8.0, 150 mM NaCl, 0.2% (v/v) Nonidet P-50 and 2 mM DTT, and the proteins purified by nickel affinity chromatography. Spin-labeling of the C633E mutant was performed by incubation with a five-fold molar excess of 3-(2-Iodoacetamido)-proxyl free radical dissolved in methanol over night at 4°C. Free spin-label was removed by buffer exchange or SEC into 20 mM Na_2_HPO_4_ pH 6.5 and 150 mM NaCl. Nuclear magnetic resonance (NMR) spectra were acquired on Bruker Avance III 600 and 800 spectrometers with a cryogenic triple resonance probe and a Bruker Avance III 700 with a triple resonance probe at concentrations of 0.1–0.5 mM in the same buffer at 300 K. Data where processed with NMRPipe (Delaglio et al., 1995) and analyzed using NMRView (Johnson and Blevins, 1994). The transfer of backbone assignment from the wt protein (Dietl et al., 2014) was confirmed by analyzing HNCA, HNCACB and CBCA(CO)NH spectra of the C633E mutant. Chemical shift based secondary structure predictions and structure based chemical shift predictions where done using the programs TALOS+ (Shen et al., 2009) and SPARTA+ (Shen and Bax, 2010). Model-free Liparai-Szabo parameters derived from the ^15^N relaxation data of the C633E mutant were analyzed and compared to hydrodynamic diffusion tensors using the programs ROTDIF and ELM (Berlin et al., 2013). Paramagnetic Relaxation Enhancements where measured and analyzed as described (Simon et al., 2010). SAXS measurements were carried out at the BM29 beamline at ESRF in Grenoble (Pernot et al., 2013). Samples were measured in NMR buffer (20 mM Na_2_HPO_4_ pH 6.5, 150 mM NaCl, 2 mM DTT) at concentrations between 0.25 and 6 mg/mL, a temperature of 300 K and a wavelength of 1 Å. Data was processed using the ATSAS suite (Petoukhov et al., 2012).

### Pull-Down Experiments

The coding sequence for full-length human Fe65 was inserted into the pUKBK vector system (Kohli et al., 2012) by standard cloning techniques in order to attach either a streptavidin-binding peptide (SBP) together with a myc-tag or a mCherry (mChe)-tag to the protein N-terminus. Thereof, the following deletion constructs were generated: ΔPTB2 (Fe65(1-532)-(665-710)), ΔWW (Fe65(1-253)-(286-710)), and ΔWW-ΔPTB2 (Fe65(1-253)-(286-532)-(665-710)). After transfection with Lipofectamine 2000 (ThermoFisher Scientific) for 22 h, HEK293 cells were lysed in homogenization buffer consisting of 140 mM KCl, 20 mM HEPES pH 7.2, 10 mM NaCl, 5% (v/v) glycerol, 2 mM MgSO_4_, 1% (v/v) Triton-X100, 2 mM DTT, EDTA-free Protease-Inhibitor Cocktail (Roche), and 2 mM Phenantrolen. Pull-down assays were performed with Dynabeads^®^ M-280 Streptavidin (ThermoFisher Scientific) and bound proteins were eluted with biotin and further separated on Novex™ 10%–20% Tricine Protein Gels. Antibodies used for detection were the c-myc antibody (1:1000, 9E10, Roche), mCherry antibody (1:1000, 5F8, Chromotek), and GAPDH antibody (1:5000, Meridian Life Science). ECL detection was performed with the ImageQuant LAS 4000 (GE Healthcare Life Sciences). Quantification was done on the latest exposure before saturation of the brightest band on the blot, using the ImageQuant TL software.

### Co-Immunoprecipitation

Co-Immunoprecipitation (Co-IP) experiments were performed as described before (Baumkotter et al., 2014). Briefly, HEK293 cells were transfected with pcDNA3.1 constructs containing FE65-HA, FE65-Flag or APP-myc using JetPRIME (Polyplus transfection). Twenty to twenty-two hours after transfection cells were harvested and lysed in 150 mM NaCl_2_, 50 mM Tris/HCl pH 7.5, 2 mM EDTA, 1% (v/v) NP40 and freshly added Complete Protease Inhibitor mix (Roche) for 20 min on ice. After centrifugation at 16,000× *g* for 10 min the supernatant was pre-cleared with protein A Sepharose beads (GE Healthcare). Then the supernatant was incubated over night with anti-HA agarose beads (Roche) to allow binding of HA-tagged FE65. After washing bound proteins were eluted by denaturation with SDS sample buffer at 95°C. Samples were separated on 8% Tris/glycine gels and probed via immunoblotting for HA-, Flag- and myc-tagged constructs.

### Subcellular Fractionation

Subcellular fractionation was performed according to Abcams subcellular fractionation protocol. HEK293 cells were transfected as described before. Twenty to twenty-two hours post transfection cells were resuspended in 1 mL of fractionation buffer (250 mM Sucrose, 20 mM HEPES, 10 mM KCl, 2 mM MgCl_2_, 1 mM EDTA and 1 mM EGTA with freshly added Complete Protease Inhibitor mix (Roche)) and passed 10 times through a 27 gauge needle. After differential centrifugation at 720× *g* and 10,000× *g* for 5 min and 100,000× *g* for 1 h the supernatant (cytosolic fraction) was transferred and kept on ice for further analysis. The sediment (membrane fraction) was resuspended by pipetting and pass through 10 times a 27-gauge needle. Protein concentration of membrane and cytosolic fraction was determined using the BCA assay (Sigma).

### Blue Native Gel Electrophoresis

For Blue Native Gel analysis 100 μg protein of the cytosolic and membrane fraction was diluted in 1.5 M amino caproic acid, 0.05 M Bis-Tris, pH 7, 1.25% (w/v) dodecyl maltosidase and 5% (w/v) Coomassie Brilliant Blue G250, as described in detail before (Eggert et al., 2009). Afterwards, samples were separated on a 4%–15% (w/v) Tris-HCl gel (Biorad), transferred on a PVDF membrane and probed via immunoblotting for HA- and myc-tagged constructs.

## Results

### Fe65-PTB2 Dimerization

Recombinantly expressed human Fe65-PTB2 (residues 534-667) is difficult to purify as it precipitates at higher protein concentrations in the mg/mL range. Instability is related to the exposure of a hydrophobic crevice that corresponds to the AICD binding site and complex formation dramatically enhances solubility about a 100-fold (Radzimanowski et al., 2008c). When purified via SEC, Fe65-PTB2 partitions in monomeric, dimeric and tetrameric species as validated by multi-angle light scattering (MALS) and on SDS-PAGE the protein appears as detergent-resistant dimer (Supplementary Figure S1). Unspecific aggregation of Fe65-PTB2 at concentrations in the mg/mL range can be prevented by the addition of glycerol and we subsequently crystallized the domain and solved its crystal structure by molecular replacement at 2.6 Å resolution (Table 1).

**Table 1:** **Data collection and refinement statistics**.

**Data collection**	
Space group	P 1 21 1
Cell dimensions	
a, b, c (Å)	56.6 104.3 60.6
*α, β, γ* (°)	90 112.0 90
Resolution (Å)	49.44–2.6 (2.74–2.6)
R_pim_ (%)	7.3 (39.5)
Wilson B-factor (Å^2^)	39.0
I/σ(I)	9.0 (2.3)
CC1/2	99.1 (83.5)
Completeness (%)	99.3 (98.3)
Redundancy	6.6 (6.2)
Wavelength (Å)	1.033
**Refinement**	
No. reflections	19931 (1993)
R_work_ (%)	19.3 (29.6)
R_free_ (%)*	24.1 (40.6)
No. atoms	3970
protein	3832
ligand/ion	60
Protein residues	501
B-factors (Å^2^)	50.4
R.m.s. deviations	
bond lengths (Å)	0.002
bond angles (°)	0.60
Ramachandran plot	
allowed (%)	99.8	

*for 5% of all data. Statistics for the highest-resolution shell are shown in parentheses.

Fe65-PTB2 crystallizes as dimer of dimers with a continuous central β-sheet (Figure 1C). Dimerization occurs via a structural transition of the C-terminal α-helix α3 within one Fe65-PTB2 subunit (the “complementing” subunit) in respect to the conformation as seen in the previously solved AICD/Fe65-PTB2 complex (rmsd of 0.85 Å for 123 Cα-atoms; Figure 1D, Supplementary Figure S2; Radzimanowski et al., 2008c). The last two helical turns dissolve (starting at L^656^) and adopt an extended β-conformation that complements the “accommodating” subunit *in trans* (dimer interface: 585 Å^2^). The interface is classified just about stable (Krissinel and Henrick, 2007). The newly formed β-strand (defined here as β^ct^) quasi-symmetrically mediates also the dimer of dimer contact with the tetrameric assembly being stabilized by a disulfide bridge between respective cysteine (C^661^) residues (Figure 1C).

### Fe65-PTB2 Dimer Structure in Solution

**Figure 2:**
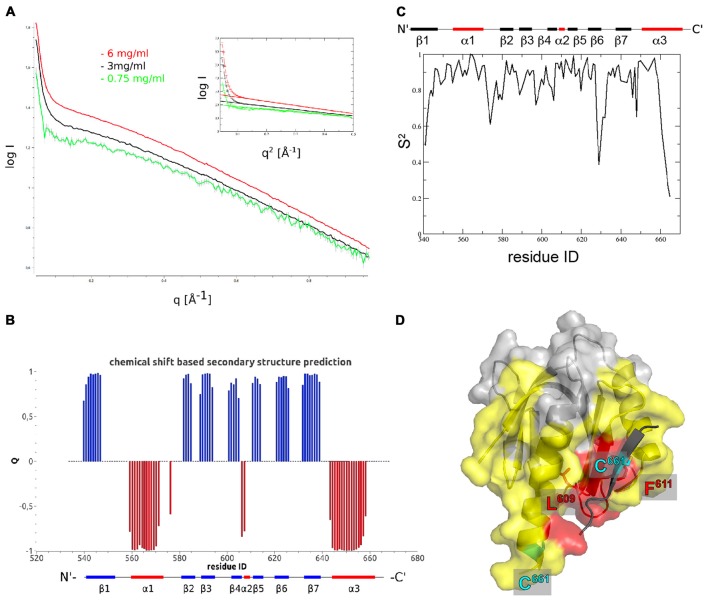
**The Fe65-PTB2 dimer in solution. (A)** Small angle scattering data measured at three different protein concentrations. The presence of self-aggregation leads to a pronounced increase in scattering intensity at low angles. The radius of gyration extracted from the Guinier plot (inset) is slightly higher than expected for a dimer and the initial intensity values almost reaches the expected value for the dimer. **(B)** Secondary structure predicted from backbone chemical shifts with positive blue bars indicating β-sheets and negative red bars α-helices. The secondary structure of the accommodating subunit (long α3 helix) is shown below for comparison. **(C)** Backbone order parameters S^2^ derived from ^15^N nuclear magnetic resonance (NMR) spin-relaxation data. The decrease of the order parameter for the C-terminal residues indicates the unfolding of the α-helix in this region resulting in a rapid reorientation of the N-H bond vectors on a ns to ps timescale. **(D)** Residues experiencing paramagnetic relaxation enhancements at the backbone NH when a nitroxide spin-label is attached to C^661^. The protein surface of the complementing subunit is shown in color if the average intensity ratio of the observed ^1^H-^15^N peak in the paramagnetic and diamagnetic NMR spectra of the corresponding residue and its two neighbors is smaller than 0.7 and thus identifies amino acids that are close to the paramagnetic center. Residues in yellow are bleached for molecules that are simultaneously ^15^N and nitroxide labeled, while residues in red are also bleached when exclusively ^15^N and nitroxide labeled proteins are mixed. The spin-label carrying C^661^ residues are highlighted for the monomer (on the C-terminal α-helix) and the crystallographic dimer (on the extra β-strand).

Having solved the crystal structure of Fe65-PTB2, we had to make sure that the observed interactions did not represent a crystallographic artifact and are also present in solution. We therefore first performed concentration dependent (0.25–6 mg/mL) small angle X-ray scattering (SAXS) measurements under reducing conditions to avoid the covalent and likely non-physiological cysteine bridge. The data showed a sharp increase in intensity at very small scattering angles that becomes more pronounced with higher concentrations and thus confirming the observation of the presence of aggregation (Figure 2A). Accordingly, the deduced molecular masses showed a strong concentration dependence that reflects the monomer-dimer transition. Calculating the theoretical scattering curves of the monomer, dimer and tetramer structures and fitting them against the experimental data, revealed the best fit to correspond to the crystallographic dimer (Supplementary Figure S3A), which holds true for the whole concentration range and also when the data are interpolated to zero concentration. Calculations of monomer and dimer content based on fitting linear combinations of two structures range from more than 20% of monomer to almost exclusively dimer at higher concentrations, but should be taken as rough estimates with the given data quality and the insecurity of especially the dimer structural model. In accordance with these data, a dissociation constant could be estimated by preliminary isothermal titration calorimetry (ITC) measurements to be in the low micromolar range (data not shown).

In order to obtain high resolution structural information for Fe65-PTB2 dimerization in solution, we performed an extended NMR characterization. Overall, we observe a high consistency between the backbone chemical shift data and the dihedral angles observed in the crystal structure (Figures 2B, Supplementary Figure S3B). For the C-terminus, the chemical shifts predict the existence of a helix until Y^658^ and indicate an increase in backbone flexibility starting from M^655^. Interestingly, the observed secondary Cα-Cβ chemical shift differences for the C-terminus are in between the values predicted for the accommodating (long C-terminal α-helix) and complementing (β-sheet augmentation) subunits of the crystal structure.

A more detailed picture for the internal dynamics and dimerization was obtained by the analysis of ^15^N relaxation data. The average ratio of transverse and longitudinal relaxation rates measured at 300 K indicated a rotational correlation time τ_c_ of 10.6 ns. This value compares to 8.9 ns (for complementing subunit) and 15.7 ns (for crystallographic dimer) as calculated from the coordinates. Assuming a rapid exchange between rigid monomers and dimers the experimental value would suggest a high percentage of monomers in solution. However, since the intermolecular interaction is mediated by the flexible C-terminus, we envision a dimer with a rather flexible connection between the monomers and thus with faster effective rotational correlation time than expected for a rigid dimer. This model is supported by the observation of quickly reducing backbone order parameters S^2^ for the C-terminal residues starting from Y^658^ (Figure 2C, Supplementary Figure S3C).

To further characterize the oligomerization in solution, we introduced nitroxide spin-labels covalently attached to cysteine residues to measure paramagnetic relaxation enhancements (PREs). The presence of the electron spin leads to signal broadening of nuclear spins in spatial proximity (less than ~20 Å) to the nitroxide and can assist NMR protein structure determination. Due to the *r*^−6^ dependence of the induced relaxation, the signal bleaching can also be used to structurally and dynamically characterize specific encounter complexes (Clore, 2015). Since Fe65-PTB2 contains six native cysteines and the evaluation of the experiment requires a single spin-label attached to each molecule, we performed an extensive mutational analysis to determine the accessibility and structural importance of all native cysteines. In the end, only two cysteines (C^633^ and C^661^) were solvent exposed to be efficiently paramagnetically labeled. Of particular interest are the PRE results for the C633E mutant, which positions the spin-label solely on C^661^ at the C-terminus in the center of the oligomerization region. We measured the intensity ratios in ^15^N-^1^H heteronuclear correlation spectra (HSQC) between the paramagnetic and diamagnetic state of the molecule (Supplementary Figures S3D,E). Due to the instability and precipitation of the molecule in solution during the measurements, a number of intensity ratios larger than one for residues that are not in proximity of the nitroxide were observed. Therefore, and because of the difficulties to accurately model the spin-label being attached to a flexible C-terminus, we resign from a detailed quantitative analysis of the data. A qualitative picture however can be obtained by plotting the experimental I_para_/I_dia_ ratios onto the X-ray structure. The lowest ratios are observed for residues in the C-terminal helix and the loops and secondary structure elements in the vicinity of the C-terminus. To disentangle intra- and inter-molecular contributions, we performed a second experiment with a mixed sample of ^14^N-paramagnetic and ^15^N-diamagnetic molecules. The observed PREs are exclusively due to inter-molecular proximity of the radical. Bleaching was observed for patches adjacent to the hydrophobic crevice and on surface loops consistent with the presence of the dimer and tetrameric species in solution (Figure 2D). Strongest bleaching with I_para_/I_dia_ ratios below 0.3 occurred for residues L^609^ and F^611^ that also are in closest contact within the crystallographic dimer and for C^661^ itself that also bridges the observed dimer of dimers.

Taken all NMR measurements together, a transient dimer formation as seen in the crystal structure is validated as homotypic interaction in solution. The tetrameric and covalent linkage of two dimers seems to be favored only under high concentrations and oxidizing conditions as seen in the crystallographic array.

### Fe65-PTB2 Mimics the AICD

**Figure 3:**
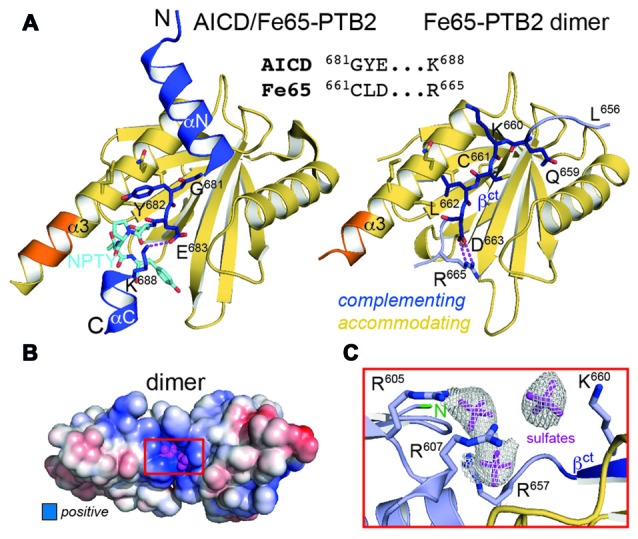
**Fe65 mimicry of AICD binding. (A)** Left: X-ray structure of the AICD/Fe65-PTB2 complex (PDB: 3dxc; Radzimanowski et al., 2008c). The central interacting part of the AICD is detailed: G^681^YE in dark blue, N^684^PTY in cyan. Right: same view and coloring of the Fe65 dimer with the AICD replaced by the accommodating subunit. The geometry and type of interactions mimic the AICD/Fe65-PTB2 complex. Matching sequences are given in the alignment. Coloring as in Figure 1D. **(B)** Surface potential (±5 *k_B_*T/e; blue: positive, red: negative) of the Fe65-PTB2 dimer. Dimerization results in an extended positively charged groove with tightly bound sulfate ions originating from crystallization. **(C)** Coordination and electron densities (2mF_o_-DF_c_, 1.0 σ) for the centrally bound sulfate ions (magenta). Binding occurs next to strand β^ct^ and the N-terminus of Fe65-PTB2 (green). Same orientation as in Panel **B** as indicated by the red rectangle.

The central part within the AICD/Fe65-PTB2 interface has been previously shown to be constituted by antiparallel β-augmentation of the PTB domain with the G^681^YE sequence fingerprint of the AICD (APP695 numbering; Figure 3A, left panel; Radzimanowski et al., 2008c). The glycine presents an essential hinge that places the N-terminally located helix αN of the AICD almost perpendicular to the C-terminal helix α3 of Fe65-PTB2 whereas the tyrosine residue (Y^682^) is imbedded in a hydrophobic pocket formed by residues of helix α3. Glutamate E^683^ is involved in an intramolecular salt bridge with a lysine (K^688^) following the NPTY^687^ sequence. In the crystal structure of the Fe65-PTB2 dimer, the induced strand β^ct^ with the C^661^LD sequence directly matches to the AICD strand (Figure 3A, right panel). Cysteine C^661^ occupies the glycine position although due to the restrained main chain flexibility it does not introduce a similar hinge. The hydrophobic leucine L^662^ superposes with the tyrosine and aspartate D^663^ forms an AICD-equivalent intramolecular salt-bridge with arginine R^665^. Thus, the complementing Fe65-PTB2 mimics the interacting AICD in space and charge. Of note, the accommodating Fe65-PTB2 subunit does not show the structural transition. The hydrophobic crevice of the complementing subunit is therefore still available, however, the adjacent C-terminal binding site for helix αC of the AICD is destroyed by the helical unwinding and the respective space is occupied by the accommodating subunit (Supplementary Figure S2C). In summary, Fe65-PTB2 dimerization results in a structural change that blocks the AICD binding site either fundamentally in the accommodating subunit or partially in the complementing subunit.

### A Basic Cluster Next to the Dimerization Site

In order to evaluate changes of the surface properties due to dimerization we calculated surface charge potentials. The analysis revealed a pronounced positively charged patch (R^605^, R^607^, R^657^, K^660^ and R^665^) in the center of the dimer directly located at the transition site of the C-terminal helix (Figures 3B,C). Due to its location, the shape of the patch differs between an extended (complementing subunit with extended strand β^ct^) and a condensed form (accommodating subunit with folded C-terminal helix; Supplementary Figure S4). Fe65-PTB2 was crystallized in sulfate conditions and we find sulfate ions bound to both the condensed and extended patches. Most strikingly, in the elongated patch next to the dimer interface we find three adjacent sulfate ions (Figures 3B,C). The spatial arrangement of the ions perfectly match to the three phosphoryl-groups of the head-group (IP_3_) of phosphatidyl-inositol-4,5-bisphosphate (PIP_2_; Supplementary Figure S4), which has been found earlier to bind to Fe65 by liposome flotation assays (Cao and Südhof, 2004). PIP_2_-binding is a recurrent and functionally important feature of many PTB domains due to their juxtamembrane location and always occurs in similar basic clusters (Uhlik et al., 2005). Of note, also the N-terminus of Fe65-PTB2, and thus the PTB1-PTB2 linker region implicated in the intramolecular closure by binding to the WW-domain (Cao and Südhof, 2004), locates next to the basic cluster.

### Fe65 Dimerization *In Vivo*

**Figure 4:**
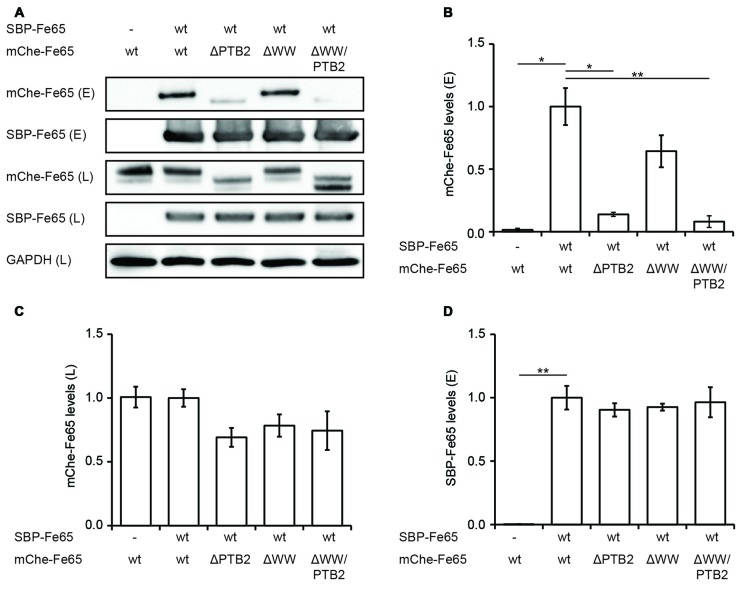
**Deletion of the PTB2 domain impairs Fe65 dimerization in cells. (A)** HEK293 cells expressing streptavidin-binding peptide (SBP)-myc-Fe65 (SBP-Fe65) and mCherry-Fe65 (mChe-Fe65) as wildtype (wt) or deletion constructs were subjected to pulldown analyses. Total cell lysates (L) and eluates (E) were analyzed with antibodies against myc, mCherry and GAPDH. **(B)** Levels of co-precipitated mChe-Fe65 constructs in the eluate are significantly reduced in both constructs harboring a deletion of the PTB2 domain. **(C)** Confirmation of similar levels of mChe-Fe65 in the lysate. **(D)** Similar amounts of SBP-Fe65 are eluted in all experiments. No GAPDH signal is seen in the eluate (not shown). Mean ± SEM of *n* = 3 are shown (*p < 0.05, **p < 0.01, *t*-test).

All structural studies have been performed *in vitro* with isolated Fe65-PTB2 at rather high protein concentrations and they do not necessarily reflect the *in vivo* situation in context of the full-length protein and the cellular environment. We therefore set out to determine its relevance by testing Fe65 dimerization in the cellular context. HEK293 cells expressing Fe65 full-length protein fused N-terminally to a SBP and deletion variants missing either the WW domain (Fe65ΔWW), the PTB2 (Fe65ΔPTB2) domain or both (Fe65ΔWW/PTB2; Figure 4), were subjected to streptavidin-based isolation. Indeed, all precipitates of SBP-tagged Fe65 also recovered mCherry-tagged Fe65 in the eluate, and thus proving Fe65 dimerization in a cellular context (Figures 4A,B). Deletion of exclusively the PTB2 domain resulted in a strong reduction of the interaction with full-length Fe65 and the same was true for a Fe65 deletion mutant lacking the PTB2 and WW domains. In contrast, deletion of solely the WW domain did not significantly interfere with Fe65 dimerization. The negative control of the input of SBP- and mCherry-tagged Fe65 validates the dimerization event (Figures 4C,D). These results show that Fe65 dimerization takes place in a cellular environment and implement the PTB2 domain being mainly responsible for dimer formation.

**Figure 5:**
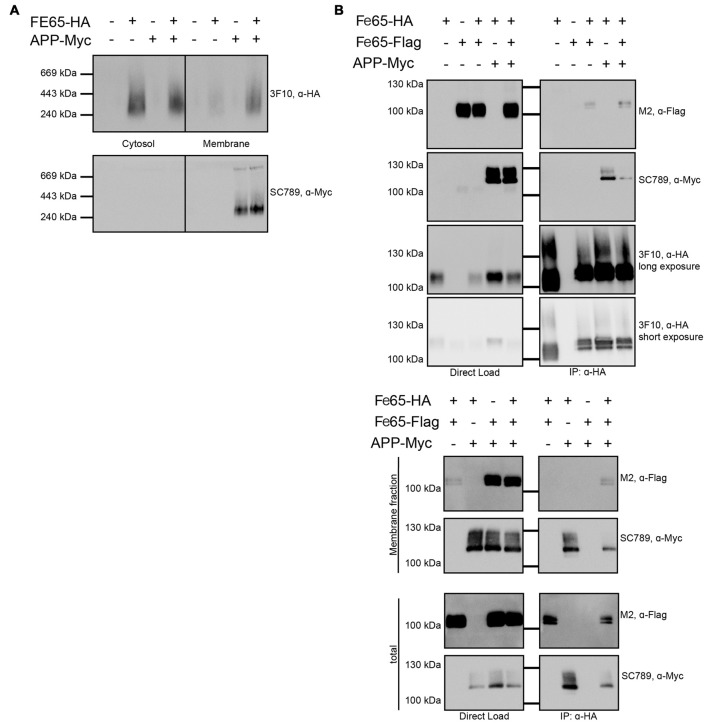
**Influence of APP on Fe65 dimerization**. HEK cells expressing exogenous Fe65-HA, Fe65-Flag or APP-myc were subjected for subcellular fractionation. **(A)** The cytosolic and membrane fractions were separated on a BlueNative-Gel and analyzed by Western Blotting using 3F10 (anti HA) and SC789 (anti myc) antibodies. Note the shift of Fe65 from the cytosolic to the membrane fraction when co-expressed with APP. **(B)** Co-Immunoprecipitation (Co-IP) analysis of whole cell lysates (upper panel) and whole cell lysates and membrane fraction (lower panel) of HEK293 cells expressing Fe65-HA and Fe65-Flag alone or together with APP-myc. Cells expressing Fe65-HA and APP-myc served as positive and cells lacking Fe65-HA as negative control. For direct load 4% of the total extracts were loaded. Immunoprecipitation was carried out with anti-HA antibody covered beads. Immunoprecipitates were eluted by denaturation and probes were subjected for PAGE (8% Tris/glycine gels) and Western analysis using 3F10 (anti HA), SC789 (anti myc) and M2 (anti Flag) antibodies.

Furthermore, we tested via Blue Native Gel analyses, if Fe65 migrates as a dimer. The analyses revealed a single band with a molecular weight of about 200 kDa pointing indeed to a full-length Fe65 dimer (Figure 5A). In HEK cells, Fe65 partitions into a major cytosolic and a minor membrane-bound fraction, whereas co-expression of APP caused a strong repartitioning of Fe65 towards the membrane fraction. Co-expression of APP did not alter electromobility of Fe65 in the native gel analysis. However, as APP and Fe65 have very similar molecular weights, the native gel analysis does not allow for differentiating homotypic from heterotypic complexes. In the next step we tested, if APP co-expression might affect Fe65 dimerization. For this purpose, we analyzed HEK293 cells expressing Flag- and HA-tagged Fe65 and myc-tagged APP and performed co-immunoprecipitation studies with anti-HA antibodies from total cell extracts (Figure 5B). For control we used cells expressing Flag-Fe65 and myc-APP only. The analyses revealed interactions of HA-Fe65 with both Flag-Fe65 and myc-APP. No clear reduction was observed for HA-Fe65/Flag-Fe65 interaction upon co-expression with myc-APP. However, these data again did not allow for differentiating between a trimeric complex of APP with dimeric Fe65 and two separate dimeric complexes either consisting of HA- and Flag-tagged Fe65 or HA-Fe65 and myc-APP. Therefore, we repeated the Co-IP of Fe65-HA, Fe65-Flag and mycAPP from HEK293 cell extracts from the membrane fraction. In this fraction only minor Fe65 amounts are present and we could not detect any Fe65 dimer. Upon co-expression of APP, Fe65 was shifted into the membrane fraction as expected from the known and strong APP-Fe65 interaction (Radzimanowski et al., 2008c). Interestingly, under these conditions we succeeded to precipitate the two differently tagged Fe65 molecules (HA and Flag) and APP (Figure 5B). These data show that Fe65 at least to some extend can still dimerize in presence of APP and even a trimeric species might be formed.

## Discussion

Fe65 is a versatile protein-adaptor with an interactome list of increasing size and complexity. It participates in various neuronal processes, including neurogenesis, neuronal migration and positioning, neurite outgrowth, synapse formation and plasticity, and finally in learning and memory (McLoughlin and Miller, 2008; Minopoli et al., 2012; Strecker et al., 2016). The most studied function concerns the gene transactivation complex together with APP and the histone acetyltransferase Tip60, although the pathway that at least in parts parallels Notch signaling and its gene targets are far from being understood (Cao and Südhof, 2001; Pardossi-Piquard and Checler, 2012). However, it is possible that in ageing and sporadic Alzheimer’s disease there is an increase of nuclear signaling concomitant with amyloidogenic processing of APP and the accumulation of the Aβ-peptide (Fukumoto et al., 2002; Yang et al., 2003; Goodger et al., 2009). Inline, it was found that an alternate splice variant of Fe65 (Fe65a2 isoform) lacking the last exon confers resistance against very late onset of AD (Hu et al., 2002). The exon codes for residues starting at the C-terminal end of helix α3 of Fe65-PTB2 and therefore is impaired in AICD binding. Soon after the first description of the signaling pathway, it was found that complex formation with APP includes a membrane-associated initiation process that enables Fe65 to act as transactivator of transcription once the AICD is cleaved-off (Cao and Südhof, 2004). This process was associated with an opening of Fe65 by the release of a WW-PTB2 domain interaction eventually triggered by a membrane-associated factor.

The AICD/Fe65-PTB2 contact is of hydrophobic character and recombinant expressed Fe65-PTB2 is aggregation prone (Radzimanowski et al., 2008a). Here we show by X-ray crystallography and extended NMR measurements including spin-labeling PRE techniques that homotypic dimerization of the Fe65-PTB2 domain mimics AICD binding and effectively shields the hydrophobic surface. The shielding may reflect the physiological need of chaperoning this surface in case the binding partner is not present or binding is to be prevented for functional reasons. This intermolecular protection does not contradict the predicted intramolecular WW-PTB2 interaction, which involves the PTB1-PTB2 boundary and could occur at the same time inhibiting downstream signaling via the WW-domain (Cao and Südhof, 2004). Interestingly, the interaction of the Fe65 WW domain and full length Fe65 is inhibited by excess of AICD, indicating that AICD binding to the PTB2 domain affects the interaction of PTB1-PTB2 boundary with the WW domain (Cao and Südhof, 2004). Homotypic dimerization might also impact pathological pathways including the AICD/Fe65 interaction. Of note, the Fe65a2 isoform conferring very late onset AD resistance (Hu et al., 2002) lacks the dimerization sequence and thus excludes the self-association. However, all interactions of Fe65 distinct to the dimerization site and independent of APP binding are likely to be unaffected by the homotypic Fe65-PTB2 interaction.

We demonstrate by co-immunoprecipitation assays performed in transfected HEK293 cells in the presence of Fe65/APP overexpression that at the membrane a Fe65-dimer complex still co-exists with APP, which could correlate with the previously described Fe65-activating state of a “primed complex” (Cao and Südhof, 2004). While there is no indication yet for an additional membrane-associated protein factor, activation seems to be guided by the lipid PIP_2_, which plays an important role in many endocytic events. PIP_2_-binding most likely occurs via the epitope identified by multiple sulfate ion binding in our dimeric Fe65 crystal structure and/or via Fe65-PTB1 (Radzimanowski et al., 2008b). As the epitope is in direct proximity to the dimer interface, membrane association could also have a direct influence on the monomer-dimer equilibrium. As also the PTB1-PTB2 linker region is directly adjacent, the WW-domain is likely be involved in this process as also indicated by our pull-down assays, which show at least some influence of the WW-domain on Fe65 dimerization. The WW-domain recognizes polyproline stretches (Meiyappan et al., 2007) and might bind to two proline residues close to Fe65-PTB2 and therefore to the PIP_2_-epitope. Inline, it had been found that the AICD and the WW-domain cannot bind simultaneously to the PTB-domains including the linker (Cao and Südhof, 2004).

**Figure 6:**
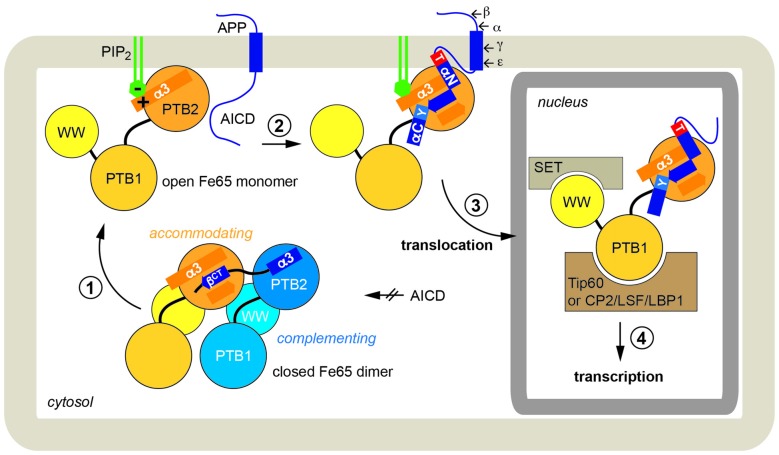
**Physiological function of Fe65 dimerization**. In a schematic model Fe65-mediated APP signaling is divided into four steps: (1) In the cytosol Fe65 forms a closed dimer by mimicking the AICD and thereby shielding the binding epitope. (2) APP binding at the cell membrane, putatively induced by phosphatidyl-inositol-4,5-bisphosphate (PIP_2_)-mediated recruitment, opens the dimer and the AICD/Fe65-PTB2 interaction is formed. The AICD changes from a disordered to a structured conformation. (3) Upon secretase cleavage of APP, AICD-Fe65 signaling complexes translocate to the nucleus. (4) Respective transcription activation processes are initiated.

We therefore propose the following integrated scenario for Fe65-mediated gene transactivation (Figure 6): Fe65 is the central adaptor for APP nuclear signaling as validated earlier. Without its upstream signal, consisting of the AICD in context of membrane-associated APP, Fe65 resides in a closed conformation. This conformation occurs in the cytosol and might avoid futile cycles and ensure efficient recycling of Fe65 pools from the nucleus back to the endomembrane system or the cell membrane. The closed conformation favors homotypic dimerization via the structural transition of the C-terminal helix α3 to strand β^ct^ that performs substrate-mimicry. At the membrane, APP and potentially other protein and lipid factors like PIP_2_, induce an opening of Fe65 and the homodimer finally dissociates. Therefore, membrane association via the basic cluster and subsequent APP binding would also result in the opening and activation of Fe65. Similarly, it appears well feasible that other functions of Fe65, involving interaction via the WW-domain with Mena or via the PTB1 domain with other surface receptors such as LRP1 might also go along with changes in the Fe65 monomer/dimer equilibrium. Further research will be required to understand these processes in more detail.

Upon ε-cleavage of APP by γ-secretase, the AICD is released from the membrane into the cytosol and the Fe65-AICD complex translocates to the nucleus. Very recent results indicate that the PTB2 rather than the WW domain is important for the nuclear localization of Fe65 (Koistinen et al., 2017). Secretase cleavage is influenced by various aspects like APP cellular localization (Haass et al., 2012), APP dimerization (Winkler et al., 2015) and APP and Fe65 phosphorylation (Bukhari et al., 2016). Due to the tight and extended interaction involving 2/3 of the AICD (Radzimanowski et al., 2008c) and co-localization studies (von Rotz et al., 2004), we favor co-migration without degradation of the AICD. Fe65-PTB1 then binds to Tip60 or other transcription factors like CP2/LSF/LBP1 (Zambrano et al., 1998). The WW-domain in the open Fe65 conformation could finally engage with downstream components as found for the nucleosome assembly factor SET (Telese et al., 2005) or the AICD might interact with Med12 from the transcriptional mediator complex (Xu et al., 2011) essential for starting transcriptional activation processes.

In summary, our structural and biochemical dissection of the molecular properties of the multiprotein-adapter Fe65 reveal the details of an essential regulatory circuit of APP signaling. The importance of APP signaling in health and disease make it worth revisiting Fe65 and its different functional conformations as target for further pharmacological investigations.

## Accession Numbers

Coordinates and structure factors have been deposited at the Protein Data Bank (PDB) with accession number 5NQH.

## Author Contributions

LPF, KH, PS, SP, SE, GS, BS and KW performed the experiments. All authors analyzed the data and contributed to writing of the manuscript.

## Conflict of Interest Statement

The authors declare that the research was conducted in the absence of any commercial or financial relationships that could be construed as a potential conflict of interest.

## Footnotes

^1^http://www.pymol.org
