# Supplementary material for: Fe65-PTB2 Dimerization Mimics Fe65-APP Interaction
Source: Front Mol Neurosci. 2017 May 11;10:140. doi: 10.3389/fnmol.2017.00140 (PMC5425604; doi:10.3389/fnmol.2017.00140)
Supplement: Supplementary file 1 [file Presentation_1.pdf]

Supplementary Material for

Fe65-PTB2 dimerization mimics Fe65-APP interaction

Lukas Peter Feilen[1,†], Kevin Haubrich[1,2,†], Paul Strecker[3], Sabine Probst[4], Simone Eggert[3], Gunter Stier[1], Irmgard Sinning[1], Uwe Konietzko[4], Stefan Kins[3], Bernd Simon[2] and Klemens Wild[1,*]

[1]Heidelberg University Biochemistry Center (BZH), University of Heidelberg, Im Neuenheimer Feld 328, 69120 Heidelberg, Germany

[2]European Molecular Biology Laboratory (EMBL), Structural and Computational Biology, Meyerhofstr.1, 69117 Heidelberg, Germany

[3]Division of Human Biology and Human Genetics, University of Kaiserslautern, Erwin-Schrödinger-Str. 13, 67663 Kaiserslautern, Germany

[4]Institute for Regenerative Medicine (IREM), University of Zurich, Wagistr. 12, 8952 Schlieren/Zurich, Switzerland

[†]Both authors contributed equally

***Corresponding author**

Supplementary Figures S1 to S4

Supplementary references

A

SEC of Fe65-PTB2

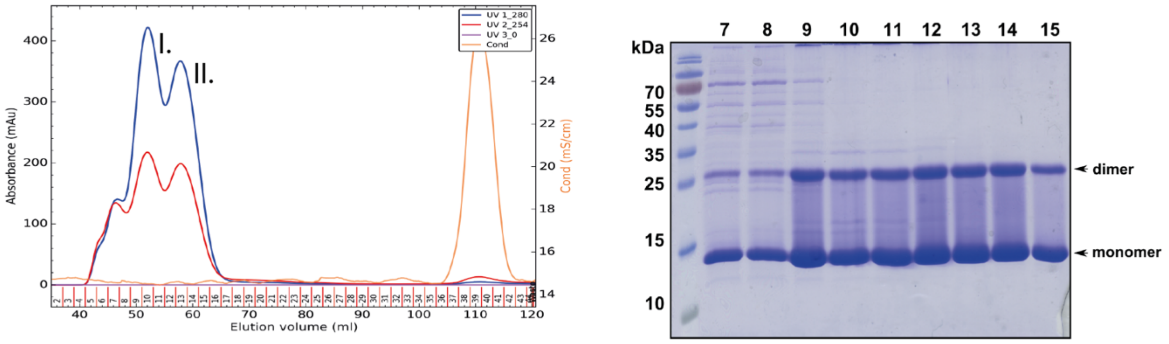

B

MALS: peak I. peak II.

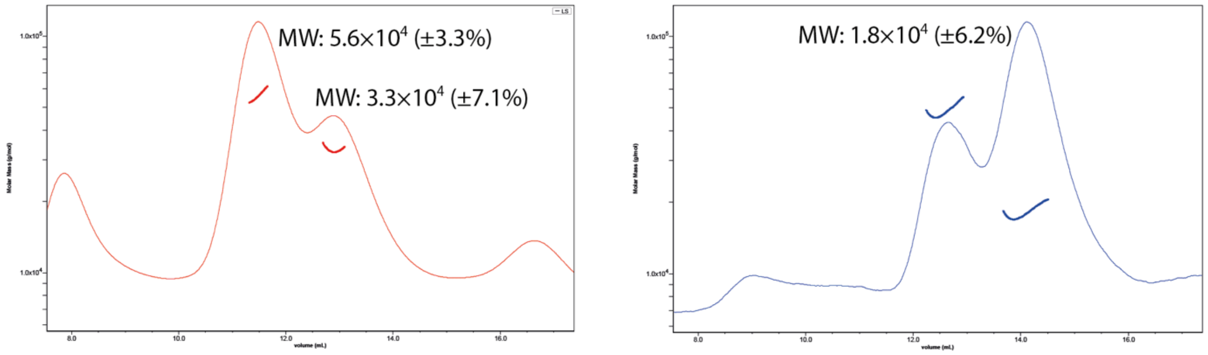

Figure S1. Biochemical evidence for Fe65 oligomerization. (**A**) Elution diagrams for size exclusion chromatography (SEC, S75 16/600) of Fe65-PTB2 and according SDS-PAGE. The protein elutes in two major peaks and appears in the gel as partially detergent-resistant dimer indicating a very hydrophobic interaction of the subunits. (**B**) Re-chromatographed peaks I. and II. (S75 10/300 coupled with multi-angle light scattering (MALS)) reveal partitioning in monomeric (MW: 18 kDa, theoretical: 15.4 kDa), dimeric (MW: 33 kDa), and tetrameric (MW: 56 kDa) species.

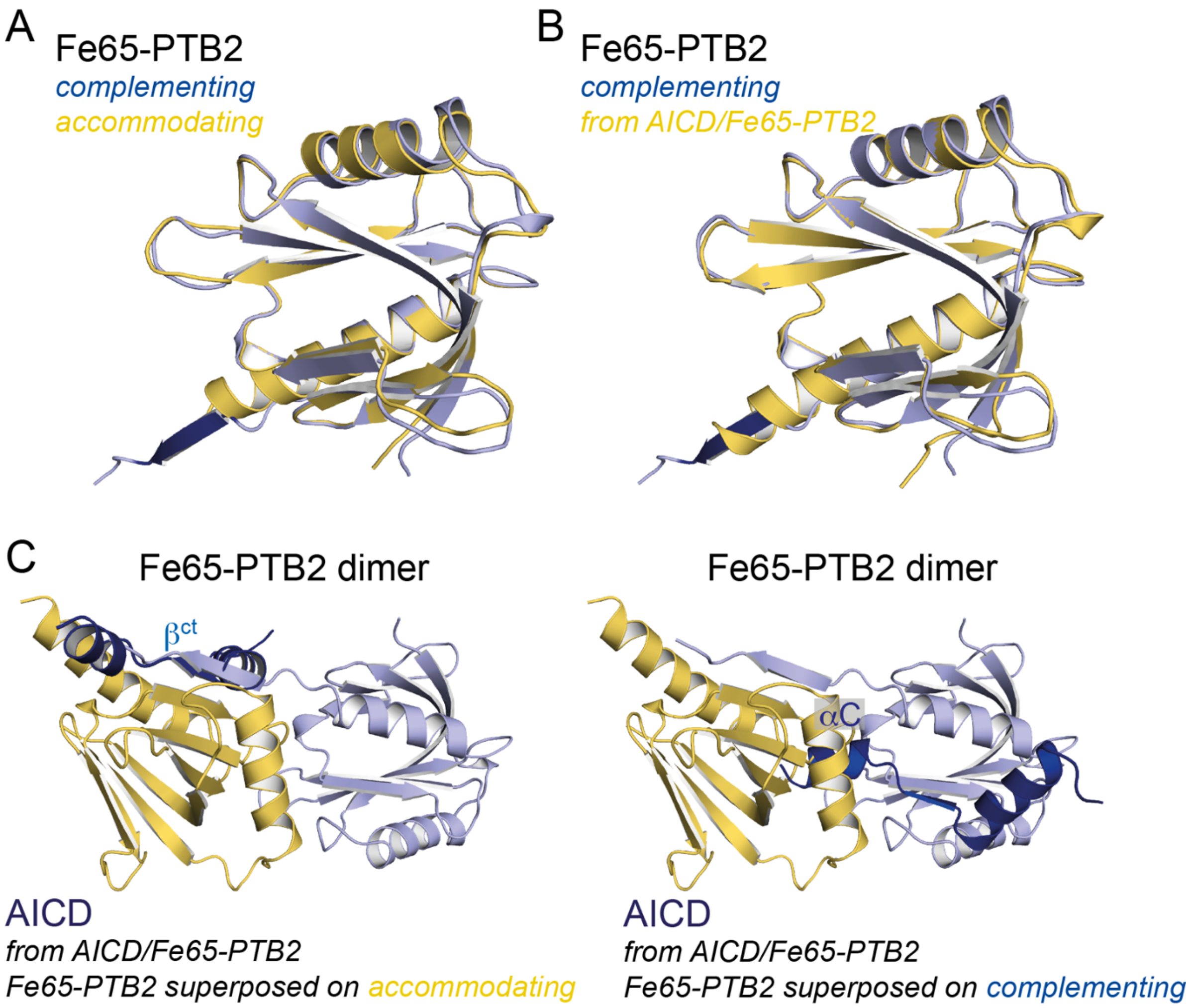

Figure S2. Structural superpositions. (**A**) The two subunits of the Fe65-PTB2 dimer. The subunits adopt identical conformations except the C-terminus. The C-terminus is flexible and in the complementing subunit forms strand β^{ct} (dark blue). (**B**) The accommodating Fe65-PTB2 subunit superposed on Fe65-PTB2 from the AICD/Fe65-PTB2 complex (PDB code 3dxc; (Radzimanowski et al., 2008)). In the complex, the C-terminal helix is fully formed and stabilized by the AICD (not shown). (**C**) The AICD and the Fe65-PTB2 dimer. Left panel: The binding site for the central β-strand of the AICD (GYE sequence) in the accommodating subunit is blocked by strand β^{ct} of the complementing subunit. Right panel: The binding site for helix αC of the AICD in the complementing subunit is blocked by the accommodating subunit. The superpositions are based on the respective Fe65-PTB2 subunits with Fe65-PTB2 (not shown for clarity) of the AICD/Fe65-PTB2 complex.

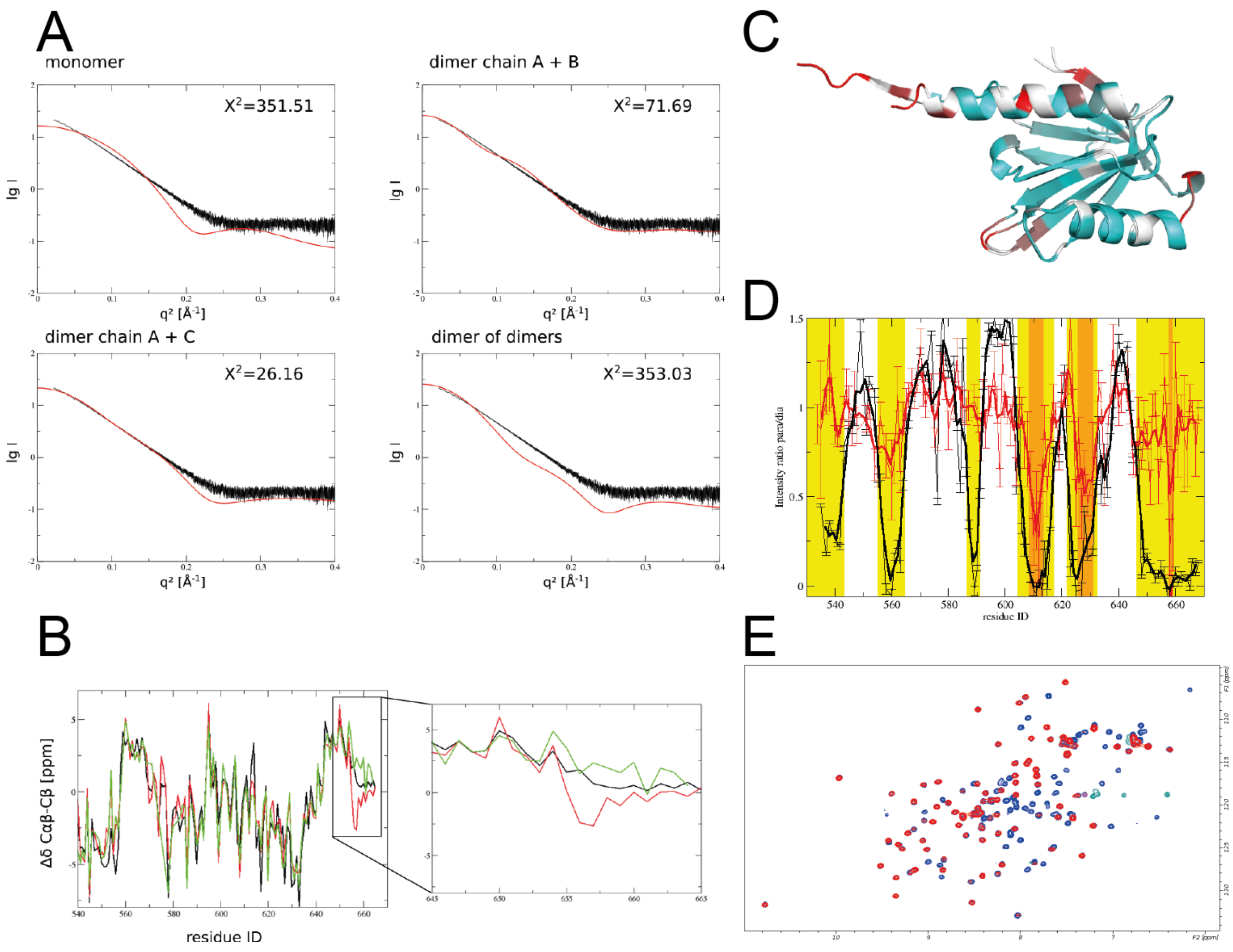

Figure S3. Biophysical characterization of the Fe65-PTB2 dimer in solution. (**A**) Fit of the experimental SAXS data extrapolated to infinite dilution to monomer (chain A, complementing), dimers (chain A+C, C: accommodating; A+B, B: alternative accommodating subunit) or dimer of dimers of the X-ray structure suggest that the protein is not monomeric in solution. The best fit is achieved for dimer (A+C). The experimental radius of gyration of 24.4±3.4 Å compares to theoretical values of 17.1 Å (monomer chain A), 23.2 Å (dimer chain A+C), 29.6 Å (dimer chain A+B) and 25.7 Å (dimer of dimers). (**B**) Secondary chemical shift differences of carbon Cα and Cβ atoms (ΔδCαβ-Cβ). The experimental values for the C-terminal helix (black) are in between the values predicted from the coordinates of the complementing and accommodating subunits. (**C**) Color representation of the ^{15}N spin-relaxation data derived backbone order parameters S^2 (Fig. 2C) on the structure of the superimposed chains A and C of the X-ray structure, with a color gradient from cyan (high S^2) to red (low S^2) and residues with no experimental data in gray. Fast timescale backbone motions are present for the N-terminus, two loop regions and the C-terminal helix. (**D**) Plot of the peak intensity ratios of ^{1}H-^{15}N HSQC spectra with reduced and oxidized nitroxide spin label for the ^{15}N/nitroxide labeled sample (yellow) and the mixed ^{15}N-only and nitroxide proteins (orange). The continuous line corresponds to a three-point average of the data points and the colored boxes correspond to the colors used for the protein surface plot in Fig. 2D. (**E**) Typical HSQC spectra of Fe65-PTB2 C633E with reduced (blue) and oxidized (red) spin labels.

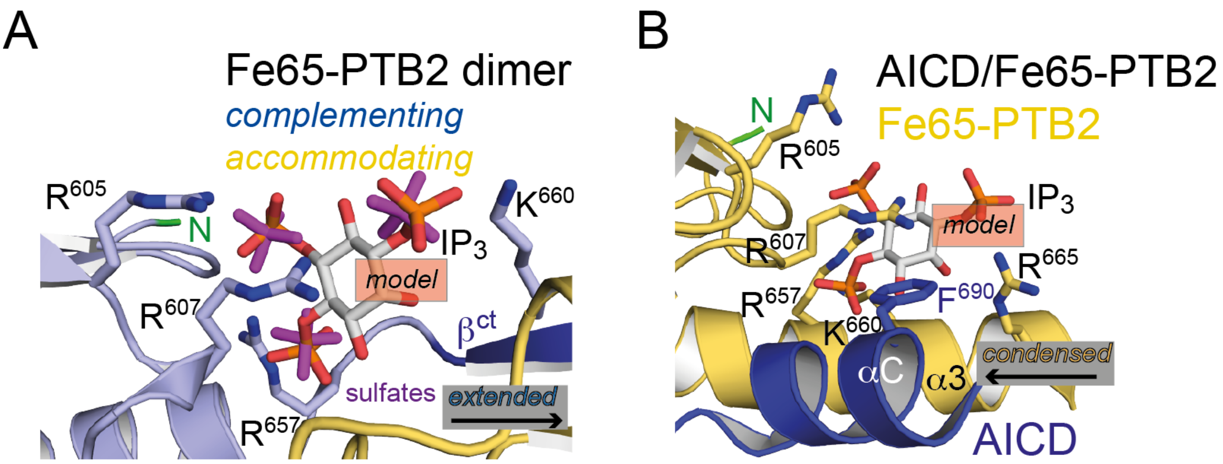

Figure S4. A basic patch for PIP_2 binding. (**A**) The spatial arrangement of three sulfate ions bound to a highly basic surface patch in the Fe65-PTB2 dimer interface perfectly match to the three phosphoryl-moieties of the IP_3 head-group of phosphatidyl-inositol-4,5-bisphosphate (PIP_2). In the complementing subunit, strand β^{ct} is part of the extended patch. Of note, also the N-terminus of Fe65-PTB2 (green) and thus the PTB1-PTB2 linker region locates next to the basic cluster. (**B**) Within the AICD/Fe65-PTB2 complex, the C-terminal AICD helix αC would complement the now condensed PIP_2-binding binding site. A solvent exposed phenylalanine (F^{690}) of the AICD would be perfectly located to interact with the inositol ring, a structural motif characteristic for protein-carbohydrate complexes (Hsu et al., 2016). Condensing of the patch results in the contribution of in total three charges (R^{657}, K^{660}, and R^{665}) from the C-terminal helix α3 of Fe65-PTB2 to the cluster. While in the extended patch arginine R^{665} is involved in the intramolecular salt-bridge in strand β^{ct} (Fig. 3a, right panel), it would contribute to PIP_2-binding in the condensed state.

References

Hsu, C.H., Park, S., Mortenson, D.E., Foley, B.L., Wang, X., Woods, R.J., Case, D.A., Powers, E.T., Wong, C.H., Dyson, H.J., *et al.* (2016). The Dependence of Carbohydrate-Aromatic Interaction Strengths on the Structure of the Carbohydrate. J Am Chem Soc *138*, 7636-7648.

Radzimanowski, J., Simon, B., Sattler, M., Beyreuther, K., Sinning, I., and Wild, K. (2008). Structure of the intracellular domain of the amyloid precursor protein in complex with Fe65-PTB2. EMBO Rep *9*, 1134-1140.
